# Geographical and temporal distribution of hawkmoth (Lepidoptera: Sphingidae) species in Africa

**DOI:** 10.3897/BDJ.9.e70912

**Published:** 2021-11-09

**Authors:** Esther N. Kioko, Alex Mutinda Musyoki, Augustine E. Luanga, Mwinzi Duncan Kioko, Esther W. Mwangi, Lawrence Monda

**Affiliations:** 1 National Museums of Kenya, Nairobi, Kenya National Museums of Kenya Nairobi Kenya

**Keywords:** hawkmoths, Sphingidae, species, diversity, distribution, Africa, National Museums of Kenya.

## Abstract

**Background:**

Hawkmoths consist of species where most adults are nocturnal, but there are some day-flying genera. Hawkmoth species have a wide variety of life-history traits, comprising species with adults (mostly nectarivorous though with some exceptions, honey-feeding), but there are also species that do not feed at all. The nectarivorous species are an important component of tropical ecosystems, with significant roles as major pollinators of both crops and wild flora with the pollination done by the adult stage. Pollinators are in decline world-wide and there is need for baseline data to provide information about their conservation strategies. Species occurrence data from Museum collections have been shown to be of great value as a tool for prioritising conservation actions in Africa. The National Museums of Kenya (NMK) have a large and active entomology collection that is in continuous growth. The NMK’s collection of hawkmoths had not been digitised prior to 2017. This moth family Sphingidae includes about 1,602 species and 205 genera worldwide (Kitching et al. 2018) with the majority of these species occurring in Africa. These moth species can also be used as indicators in biodiversity assessments as they can be easily sampled and identified. However, hawkmoths have rarely been surveyed over the long term for this purpose. Long-term datasets are of unquestionable significance for understanding and monitoring temporal changes in biodiversity. These hawkmoth data have addressed one of the most significant challenges to insect conservation, the lack of baseline information concerning species diversity and distribution and have provided key historic hawkmoth species diversity and distribution data that can be used to monitor their populations in the face of climate change and other environmental degradation issues that are facing the world today. The publication of the hawkmoth species occurrence data records in GBIF has enhanced data visibility to a wider audience promoting availability for use.

**New information:**

The hawkmoth (Lepidoptera: Sphingidae) collection at the National Museums of Kenya was digitised from 2017 – 2020 and this paper presents details of species occurrence records as in the insect collection at the NMK, Nairobi, Kenya.

The collection holds 5,095 voucher specimens consisting of 88 genera and 208 species. The collection covers the period between 1904 and 2020.

The geographical distribution of the hawkmoths housed at the NMK covers East Africa at 81.41%, West Africa at 7.20%, Southern Africa at 6.89%, Central Africa at 4.02% and North Africa at 0.2%.

## Introduction

Insect pollinators have been undergoing a decline in abundance, occurrence and diversity in many parts of the world (Biesmeijer et al. 2006, Ollerton et al. 2014, Macgregor et al. 2016). Using existing records of hawkmoths in museum and private collections over a 112-year period, Young et al. (2017) detected declines in eight species of north-eastern U.S. hawkmoth pollinators. Hawkmoth declines may have ecological effects on both the plants pollinated by these species and vertebrate predators of the moths (Young et al. 2017). Some of the plants, pollinated by hawkmoths including orchids, are rare (Sheviak and Bowles 1986, Martins and Johnson 2007) highlighting the potential conservation consequences of hawkmoth population declines especially in Africa where 70% of the species occur (Kawahara et al. 2009, Ballesteros-Mejia et al. 2013).Hawkmoths are strong flying Lepidoptera which include many pollinating species that typically feed nocturnally, but a few feed diurnally on pale-coloured flowers with long corollas and a sweet scent (Miller 1997, Johnson and Raguso 2015, Johnson et al. 2016). Increasing attention to pollinators and their role in providing ecosystem services has revealed a paucity of studies on long-term population trends of most insect pollinators in many parts of the world (Young et al. 2017, Chiquetto-Machado et al. 2018). Museums have long-term datasets of unquestionable significance for understanding and monitoring temporal changes in biodiversity and that can address one of the most significant challenges to insect conservation, the lack of baseline information concerning species diversity and distribution (Summerville and Crist 2003, Lampe and Striebing 2005). Though hawkmoths play important roles in the ecosystem in pollination and as indicator species, data on their diversity, temporal and geographic distribution in Africa is limited. The National Museums of Kenya have a large active entomology collection that is in continuous growth (Kioko et al. 2020). The NMK’s collection of hawkmoths had not been digitised prior to 2017 (Kioko et al. 2021). This project was undertaken to excavate data from the NMK collection to avail data on the spatial and geographical coverage of hawkmoth species in Africa.

## General description

### Purpose

To create an online freely accessible, openly licensed resource for users.

## Project description

### Title

Digitising the hawkmoth voucher specimens housed at the National Museums of Kenya.

### Personnel

Data mining from the National Museums of Kenya collection and additional field data from the Taita Hills ecosystem that forms the northernmost Eastern Arc Mountains was done by Esther N. Kioko, Alex M. Musyoki, Augustine Luanga, Duncan Mwinzi and others. Bioinformatics support for online publication of the data was provided by Esther W. Mwangi and Lawrence Monda.

### Funding

The project is supported by the JRS Biodiversity Foundation, USA, with co-funding provided by National Museums of Kenya.

## Sampling methods

### Study extent

**Figure 1.**
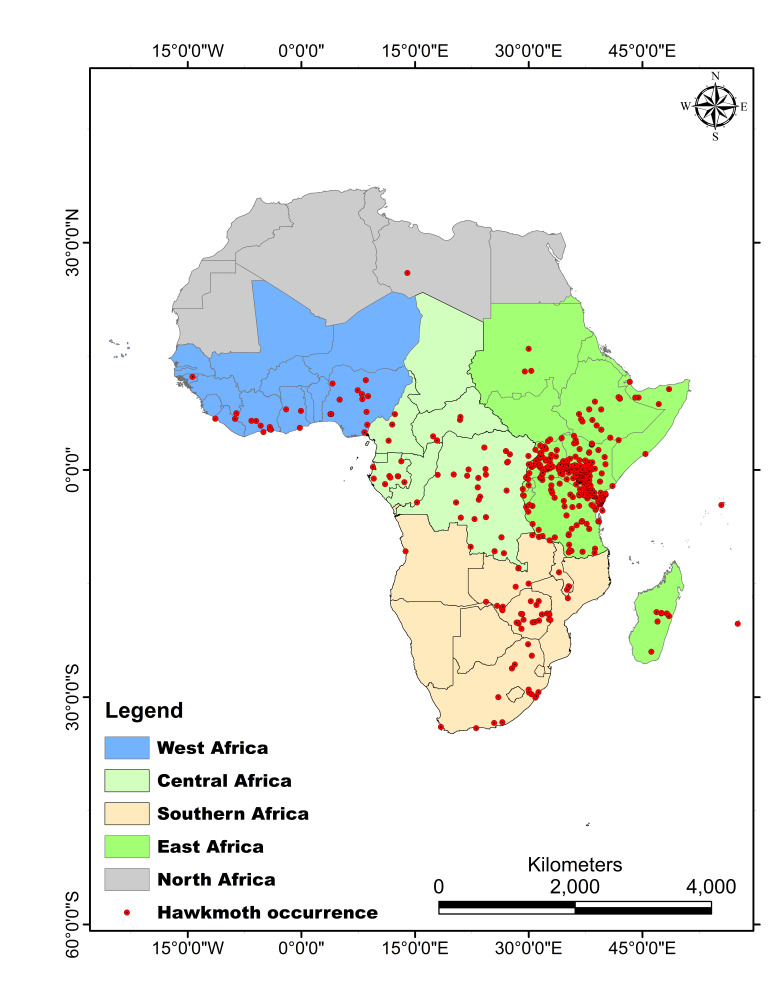
Geographic coverage of the hawkmoth voucher specimens housed at the National Museums of Kenya.

The digitised hawkmoth voucher specimens are all from Africa with several regions: East Africa at 81.41% with 4,148 records, West Africa at 7.20% with 367 records, Southern Africa at 6.89% with 351 records, Central Africa at 4.02% with 205 records and North Africa at 0.2% with one record.The spatial coverage within the five African regions is as shown in Fig. 1. The leading records for East Africa consists of 2,566 from Kenya, 829 from Uganda and 427 from Tanzania.

### Sampling description

The hawkmoth specimens, housed at the NMK Invertebrate collection, are the result of multiple field expeditions and research projects. Most of the specimens lack information on the sampling protocol and, in case a certain method was used, then it was not indicated on the specimen label. The specimens were first catalogued and pinned; they were then preserved by drying in an oven.

### Quality control

Once the specimens are brought to the invertebrate collection, taxa experts revise the associated metadata i.e. species name (taxonomy) and locality. The geographical coordinates that were lacking, as is the case with old museum specimens, were obtained using a geo-referencing web service GEOLocate (Rios 2014) by use of available textual locality data. Verification of the taxonomic names was done by checking against various references (Carcasson 1967, D'Abrera 1986, Kitching and Cadiou 2000, Kitching 2017).However, Sphingidae is a diverse family of moths and the taxonomy of the species is far from complete. There are some uncertainties in some of the identifications considering the existence of species complexes and also considering the dynamic nature of taxonomic treatments and changes in species concepts associated with the names used.

## Geographic coverage

### Description

The digitised hawkmoth voucher specimens are all from different regions within Africa as follows: East Africa at 81.41% with 4,148 records, West Africa at 7.20% with 367 records, Southern Africa at 6.89% with 351 records, Central Africa at 4.02% with 205 records, North Africa at 0.2% with one record and records not with assigned region at 0.02% with the spatial coverage as shown in Fig. 1.

### Coordinates

-35.174 and 37.44 Latitude; -17.578 and 52.383 Longitude.

## Taxonomic coverage

### Description

At the National Museums of Kenya, there are 5,095 hawkmoth voucher specimens that have been digitised and published in GBIF through the Integrated Publishing Tool (Kioko et al. 2021). The specimens comprise 243 species belonging to 88 genera, with the leading genus in occurrence records being *Temnora* at 699. Amongst the species, *Hippotioncelerio* is the most abundant with 403 records, *Agriusconvolvuli* at 210, *Leucophlebiaafra* at 137, *Euchloronmegaera* at 121, *Nephelecomma* at 107, *Hippotioneson* at 103 and *Acherontiaatropos* at 100.

### Taxa included

**Table:** 

Rank	Scientific Name	Common Name
family	Sphingidae	Hawkmoths

## Traits coverage

### Data coverage of traits

PLEASE FILL IN TRAIT INFORMATION HERE

## Temporal coverage

### Notes

**Figure 2.**
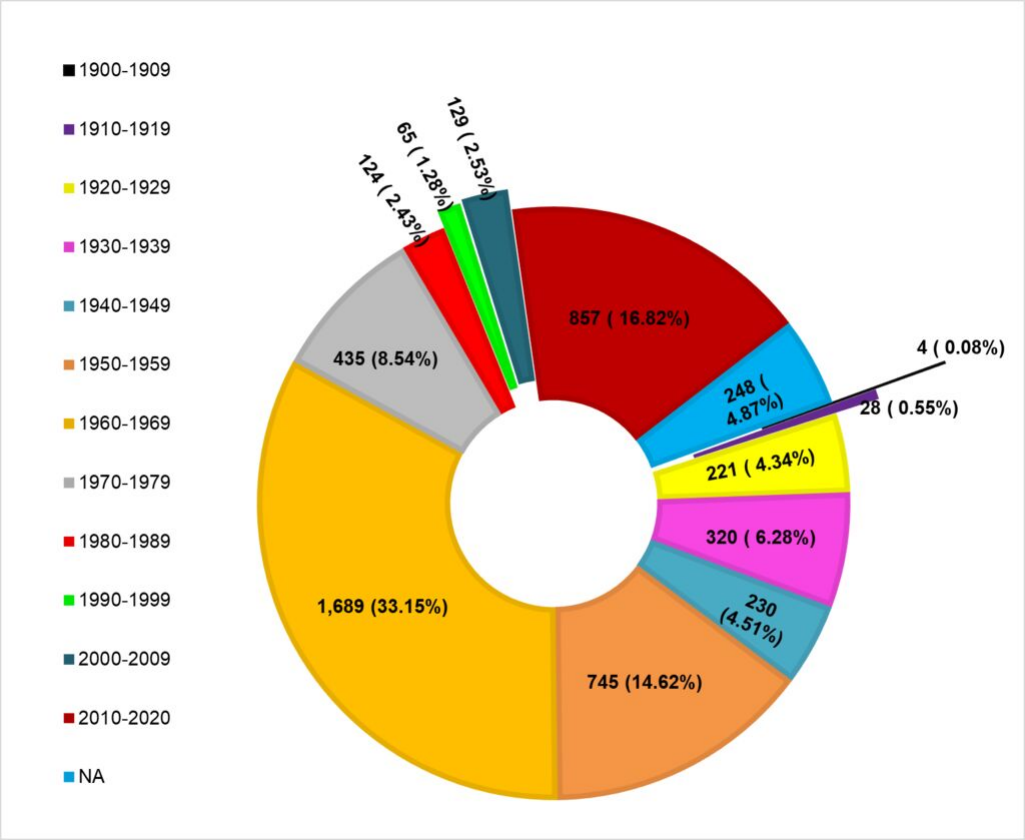
Temporal abundance of hawkmoth collection at the National Museums of Kenya.

**Figure 3.**
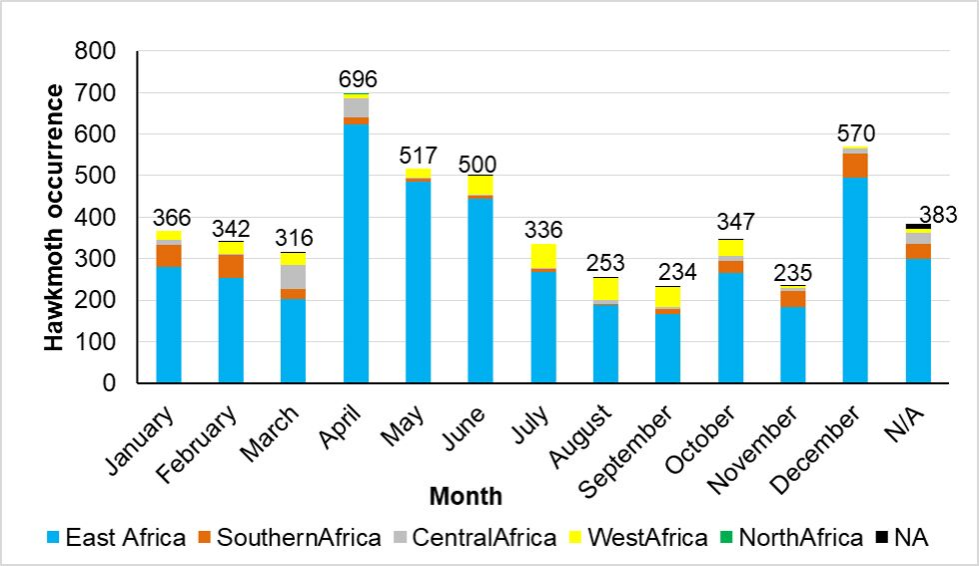
Monthly abundance of hawkmoth collection at the National Museums of Kenya.

The digitised hawkmoth collections date from 1904 to 2020. The years 1960-1964 recorded the highest values at 1,689 followed by 2010 – 2020 with 857 records, while the period 1900-1909 recorded the least at four records (Fig. 2).The voucher specimens were collected throughout the year with the highest month of collection being April with 696 records, followed by December with 570, while the months with fewest collection records were November with 235 and September with 234 (Fig. 3).

## Collection data

### Collection name

Invertebrate Zoology Section Collection, National Museums of Kenya

### Specimen preservation method

Pinned

### Curatorial unit

Species collecting event.

## Usage licence

### Usage licence

Creative Commons Public Domain Waiver (CC-Zero)

### IP rights notes

This work is licensed under a Creative Commons Attribution Non Commercial (CC-BY-NC) 4.0 Licence.

## Data resources

### Data package title

Occurrence data of hawkmoths (Lepidoptera: Sphingidae) in the National Museums of Kenya Zoological Collection

### Resource link

https://www.gbif.org/dataset/302155f9-49a6-4ee2-a01b-293067ddeeed

### Alternative identifiers

302155f9-49a6-4ee2-a01b-293067ddeeed, http://ipt.museums.or.ke/ipt/resource?r=hawkmoth_nmk_i

### Number of data sets

1

### Data set 1.

#### Data set name

Occurrence data of hawkmoths (Lepidoptera: Sphingidae) in the National Museums of Kenya Zoological Collection

#### Data format

omma-separated values (CSV)

#### Number of columns

38

#### Download URL

https://www.gbif.org/dataset/302155f9-49a6-4ee2-a01b-293067ddeeed

#### Description

This resource is a digitised format of data on the occurrence of hawkmoth species housed in the Zoology Department, National Museums of Kenya insect collection. The data provide baseline information on the distribution of different hawkmoth species and can be used for future ecology studies on hawkmoths, as well as monitoring of population trends in various habitats.

**Data set 1.** 

Column label	Column description
occurrenceID	An identifier for the Occurrence.
basisOfRecord	The specific nature of the data record.
eventDate	The date-time when the event was recorded.
year	The four-digit year in which the Event occurred, according to the Common Era Calendar.
month	The integer month in which the Event occurred.
day	The integer day of the month on which the Event occurred.
scientificName	The full scientific name, with authorship and date information, if known.
higherClassification	A list (concatenated and separated) of taxa names terminating at the rank immediately superior to the taxon referenced in the taxon record.
kingdom	The full scientific name of the kingdom in which the taxon is classified.
phylum	The full scientific name of the phylum or division in which the taxon is classified.
class	The full scientific name of the class in which the taxon is classified.
order	The full scientific name of the order in which the taxon is classified.
family	The full scientific name of the family in which the taxon is classified.
genus	The full scientific name of the genus in which the taxon is classified.
specificEpithet	The name of the first or species epithet of the scientificName.
taxonRank	The taxonomic rank of the most specific name in the scientificName.
nomenclaturalCode	The nomenclatural code (or codes in the case of an ambiregnal name) under which the scientificName is constructed.
decimalLatitude	The geographic latitude (in decimal degrees, using the spatial reference system given in geodeticDatum) of the geographic centre of a Location.
decimalLongitude	The geographic longitude (in decimal degrees, using the spatial reference system given in geodeticDatum) of the geographic centre of a Location.
geodeticDatum	The ellipsoid, geodetic datum or spatial reference system (SRS) upon which the geographic coordinates given in decimalLatitude and decimalLongitude are based.
verbatimCoordinateSystem	The coordinate format for the verbatimLatitude and verbatimLongitude or the verbatimCoordinates of the Location.
georeferencedBy	A list (concatenated and separated) of names of people, groups or organisations who determined the georeference (spatial representation) for the Location.
georeferencedDate	The date on which the Location was georeferenced.
higherGeography	A list (concatenated and separated) of geographic names less specific than the information captured in the locality term.
continent	The name of the continent in which the Location occurs.
country	The name of the country or major administrative unit in which the Location occurs.
countryCode	The standard code for the country in which the Location occurs.
locality	The specific description of the place.
type	The set of classes specified by the Darwin Core Type Vocabulary, used to categorise the nature or genre of the resource.
language	The language in which the resource is written.
institutionID	An identifier for the institution having custody of the object(s) or information referred to in the record.
institutionCode	The name (or acronym) in use by the institution having custody of the object(s) or information referred to in the record.
collectionID	An identifier for the collection or dataset from which the record was derived.
collectionCode	The name, acronym, coden or initialism identifying the collection or dataset from which the record was derived.
catalogNumber	An identifier (preferably unique) for the record within the dataset or collection.
IndividualCount	The number of individuals represented present at the time of the Occurrence.
organismQuantity	A number or enumeration value for the quantity of organisms.
organismQuantityType	The type of quantification system used for the quantity of organisms.

## Additional information

Kioko E, Musyoki A, Luanga A, Sese J, Nyangena L, Mwinzi D (2021): Occurrence data of hawkmoths (Lepidoptera: Sphingidae) in the National Museums of Kenya Zoological Collection. v.1.7. National Museums of Kenya. Dataset/Occurrence. http://ipt.museums.or.ke/ipt/resource?r=hawkmoth_nmk_i&v=1.7
